# Identification of *Fasciola* Species Using Tegumental Spines in Tissue Sections

**Published:** 2020-04

**Authors:** Arezoo FADAVI, Keyhan ASHRAFI, Hamid HASSANPOUR, Mohamad Bagher ROKNI, Seyyed Mostafa HOSSEINI, Arezoo BOZORGOMID, Leila HOSSEINPOUR, Faezeh NAJAFI, Gholamreza MOWLAVI

**Affiliations:** 1. Department of Medical Parasitology and Mycology, School of Public Health, Tehran University of Medical Sciences, Tehran, Iran; 2. Department of Medical Microbiology, School of Medicine, Guilan University of Medical Sciences, Rasht, Iran; 3. Department of Epidemiology and Biostatistics, School of Public Health, Tehran University of Medical Sciences, Tehran, Iran

**Keywords:** *Fasciola*, Tissue section, Diagnosis, Tegumantal spine

## Abstract

**Background:**

Efforts to find a reliable non-molecular means of identification has been the main purpose of the current work that always is persuaded by researchers interested in the field of parasitology.

**Methods:**

Adult fasciolids were obtained from the slaughterhouses in different parts of Iran in 2017, and investigated using the classical old fashion morphological appearances of the worms implementing a camera lucida equipped microscope. Histological procedure was subsequently performed for almost the entire collected adult worms followed by Hematoxylin and Eosin (H&E) staining technique. DNA extraction and RFLP-PCR technique were carried out for the entire fasciolid liver flukes. To attain more comparable morphological conclusions, Scanning Electron Micrographs were also implemented for two molecularly identified fasciolids.

**Results:**

Based on spine morphology observed in worm’s tissue sections two types of tegumental spines, “pointed” and “molar” shapes have been identified addressing to distinguish *F. hepatica* and *F. gigantica* species respectively. The present identification has been also supported by Molecular analysis using RFLP-PCR technique.

**Conclusion:**

There are some hidden morphological characters implemented in species identification for certain helminths. Meanwhile, the emergence of computer image analysis system (CIAS) on the scene of taxonomy, has revolutionized the accuracy of measurement in morphology by employing detailed parameters that have not been regarded before. The current study has illustrated the tegumental spines of two *Fasciola* species in tissue sections which has not been enough considered in helminthological publications so far.

## Introduction

*Fasciola* liver flukes are considered as zoonotic helminths having remarkable veterinary and public health concern in the world. *Fasciola hepatica* with worldwide distribution along with *F. gigantica* believed to be found exclusively in Africa and Asia, are responsible species causing fascioliasis (1). The high prevalence rates of human fascioliasis in a given area is not necessarily related to fascioliasis in local livestock (2).

Fascioliasis is placed among the neglected tropical diseases (NTDs) with considerable human infections while has not been received sufficient funding and enough devotion from the governmental points of view (3). Moreover economic losses due to animal fascioliasis are globally estimated above three billion US dollar annually (4). Differentiation between the *Fasciola* species has been always an attracting topic in parasitology as they demonstrate a different status of pathology, transmission pattern and epidemiology in various geographical situations (5).

To perform the needed experiments in this regard, along with the old fashion helminthological techniques for species identification, implementing of computer image analysis system (CIAS) has been successfully practiced by the interested researchers (6). Although relying on morphological characters receives less value in taxonomy nowadays, computer software mainly Image-pro plus and/or Olysia, has revolutionized the implementing of morphology in discrimination of fasciolid flukes (7) as well as similar regards towards *Echinococcus granulosus* strains (8), for instance.

Meanwhile, in critical case of genetic relationship amongst Japanese triploid forms of fasciolids in which different identities are observed in rDNA and mtDNA sequencing, the impact of molecular analysis in species identification should be significantly regarded (9). In this paper, a hidden morphological character practiced in species identification between *F. hepatica* and *F. gigantica*, has strengthened the value of morphology even at present time. Hereby, the morphology of integumental spines of fasciolids in tissue sections has been employed in species differentiation as a reliable non-molecular technique.

## Methods

Overall, 47 adult fasciolids were isolated from the cattle, goats, sheep and buffalos under the national authorized meat inspection process in slaughterhouses of Khuzestan and Kurdistan provinces in southwestern and western parts of Iran, respectively in 2017. Samples were primarily divided in two groups according to their apparent morphological features. To obtain an accurate morphological identification, the body length over the body width ratio (BL/BW) and distance between the ventral sucker and the posterior end of the body (VS-P) were considered (10). Aiming to confirm the initial grouping of the isolated worms, RFLP-PCR technique was performed (11).

### DNA extraction and PCR

A small piece of the anterior part of the body, at the level of cephalic cone, was cut and used for DNA extraction. Genomic DNA it using Bioneer DNA extraction kit (Bioneer Corporation, Daejeon, South Korea) according to the manufacturer’s instructions. PCR was performed in a final reaction volume of 25 μL using 10 μL of PCR Master mix containing 1.25 U Taq DNA polymerase, 200 μM of dNTPs and 1.5 mM MgCl2 (2 × Master Mix RED Ampliqon, Denmark); 10 pmol of each primer and 1 μL of DNA sample. Primers 5′-TTGCGCTGATTACGTCCCTG-3′ and 5′-TTGGCTGCGCTCTTCATCGAC- 3′ amplify a 680 bp target of ribosomal DNA internal transcribed spacer 1 (ITS1) gene (12). The temperature profile was an initial denaturation step at 94 °C for 10 min, followed by 25 cycles of denaturation at 94 °C for 90 sec, annealing at 58 °C for 90 sec, extension at 72 °C for 90 sec, followed by a final extension at 72 °C for 10 min. Subsequently, 3 μL of each PCR product was run on a 1.5% agarose gel and visualized using a UV transilluminator after staining with DNA Safe Stain Dye (Pishgam Biotech Co., Tehran, Iran).

RFLP reaction was carried out using RsaI restriction enzyme to distinguish specifically *F. hepatica* from *F. gigantica* (13). The digested fragments were electrophoresed on 2% agarose gel.

### Tegumental spine analysis

To implement our proposed means of discrimination, the embedded tegumental spines were primarily picked up intact from the ventral sucker area upward to the oral sucker. Subsequently, the harvested spines were photographed and measured using a camera equipped microscope with Olysia software. In the next step, tissue sections were obtained taken from the selected pieces of the almost entire isolates in 5-micrometer thickness according to conventional histotechniques. In order to attain more possibilities in a better morphological comparison, Scanning Electron Microscopy (SEM) has been also carried out for two candidate samples belonged to *F. gigantica* and *F. hepatica*.

## Results

**Fig. 1:**
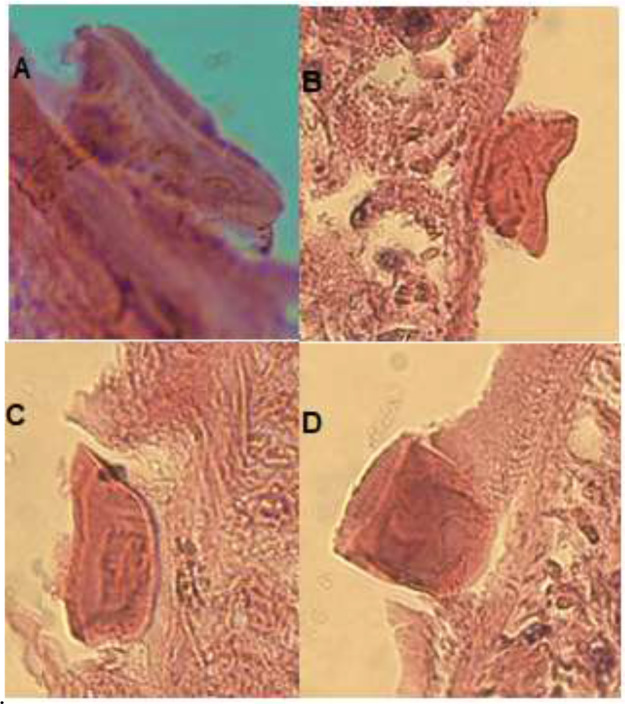
“Molar” and /or “Saddle” shape spines of *F. gigantica* (40×) on tegument in tissue sections from four different views

**Fig. 2:**
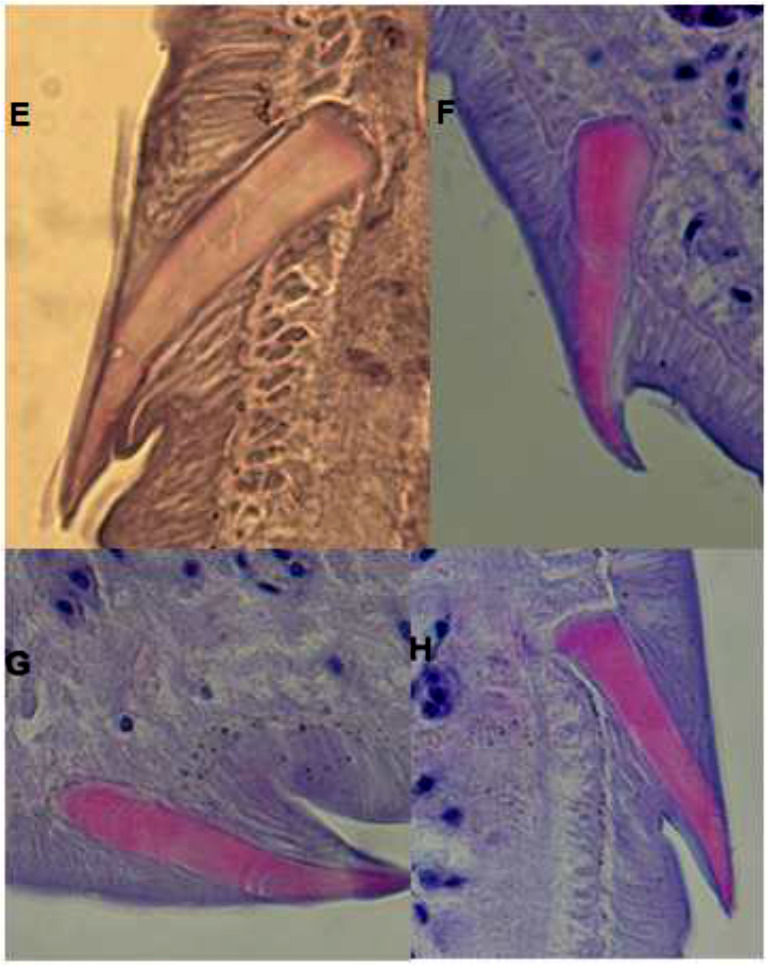
“Pointed” shape spines of *F. hepatica* (40×) seen in four different views in tissue sections

Out of the entire 47 samples, molecular studies verified 10 samples as *F. hepatica* and 37 as *F. gigantica* (Table 1). All initially recognized samples were matched based on molecular criteria. According to the collected data, the intact spines isolated from the tegument of adult worms were not characteristic for species identification due to their morphological similarity while the appearance of spines within the tissue sections were found to be discriminative in two species. The spines of *F. hepatica* were prominently pointed in shape compared with those of molar spines in *F. gigantica* (Figs. 1, 2). The measured length of the spines from the same body parts of the worms were seen significantly different in two species (*P*<0.001), with taller ones in *F. hepatica* (Table 2).

**Table 1:** PCR based differentiation of *Fasciola* species in different slaughtered animals from different geographical regions

***Host***	***Total number***	***F. hepatica***	***F. gigantica***	***Origin***
Buffalo	30	-	19	Ahvaz (Khuzestan Province)
Goat	17	-	11	Ahvaz (Khuzestan Province)
Cattle	8	-	3	Ahvaz (Khuzestan Province)
Sheep	11	-	4	Ahvaz (Khuzestan Province)
Cattle	28	10	-	Sanandaj(Kordestan Province
Total	94	10	37	-

**Table 2:** Significant difference between the lengths of the spines in two species (*P*<0.001)

***Spine***	***F. hepatica n=205***	***F. gigantica n=184***
Length	(55.68±19.4)	(29.06±13.9)
Width	(15.55±2.7)	(29.46±8.8)

## Discussion

Reporting of 2594 cases of human fascioliasis from 1970 to 1990 by Chen and Mott in 41 countries highlighted the public health concerns towards this liver fluke infection merely considered as a veterinary problem until then (14). Fascioliasis is now recognized as an emerging prevalent food-borne zoonosis worldwide (15).

Over the years, morphological characters such as body length, width, cephalic cone, and the broadness of the shoulders were used by researchers to discriminate *F. hepatica* and *F. gigantica* (16). In recent decades parallel to employing molecular techniques, as the most reliable tools for identification of adult fasciolids and intermediate forms, computer image analysis system has also been frequently used (17). In Table 1, molecularly characterized fasciolids from a high altitude region of Sanandaj in the west and those of Ahwaz, lowland in southwestern Iran is illustrated. This ecobiological sampling concerning the geographical distribution of the parasite species is in agreement with a series of works conducted by pioneers in the world (18).

Application of morphological characteristics in taxonomy of helminthes is now possible thanks to the computing software such as Image-Pro Plus which facilitates detailed measuring of morphometric parameters which are important in identification of the worms at the species level (6). Present paper is dragging the minds of the readers on tegumental spines of the fasciolid species in tissue sections for species identification, a morphological tool not received enough attention to so far. Detached spines from the tegument are not enough discriminative for species identification, compared to those are seen in tegument tissue sections. Our present look on fasciolid’s tegumental spines seems remarkable as the studied specimens were confirmed by RFLP-PCR analysis. Although this morphological points have not been clearly addressed so far, Wayne M. Meyers and colleagues had well described them in 2000 (19). In this book, they have elaborated the tegumental spines as long pointed for *F. hepatica* and flattened intracellular spins in *F. gigantica*.

To increase the presented morphological capabilities, describing of *F. gigantica* spines as “molar teeth” and /or a “saddle like “in shape, seems enough reliable from the perspective of microanatomical studies amongst *Fasciola* species. Moreover, the existence of even one pointed spine (Figs. 1, 2) in tissue sections could be in favor of *F. hepatica* and/or exclusion of *F. gigantica*.

**Fig. 3:**
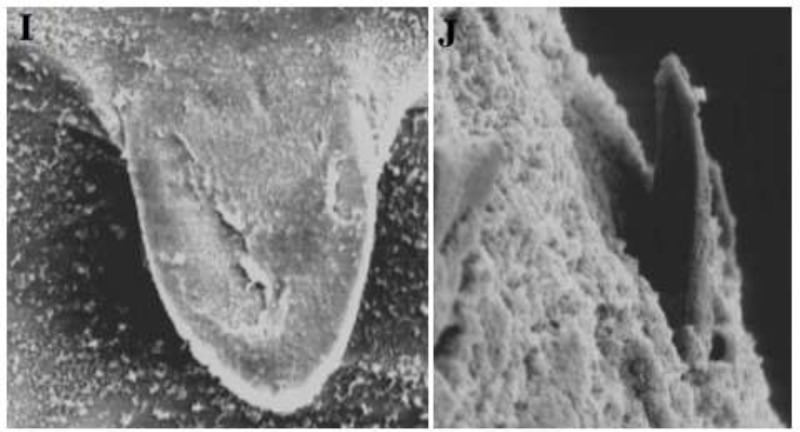
Exhibiting tegumental spines in SEM, *F. gigantica* (I), *F. hepatica* (J)

As illustrated below aiming to emphasize our present morphological tools, scanning electron microscopy (SEM) has been also carried out for two different spine models, but the results have not been satisfied (Fig. 3). Concerning the SEM experiments, previous researchers have studied on spine morphology of *F. gigantica* by which, the leaf shape rough tegument, papillae and serrated edges of the spines, were illustrated comparatively (20, 21).

**Fig. 4:**
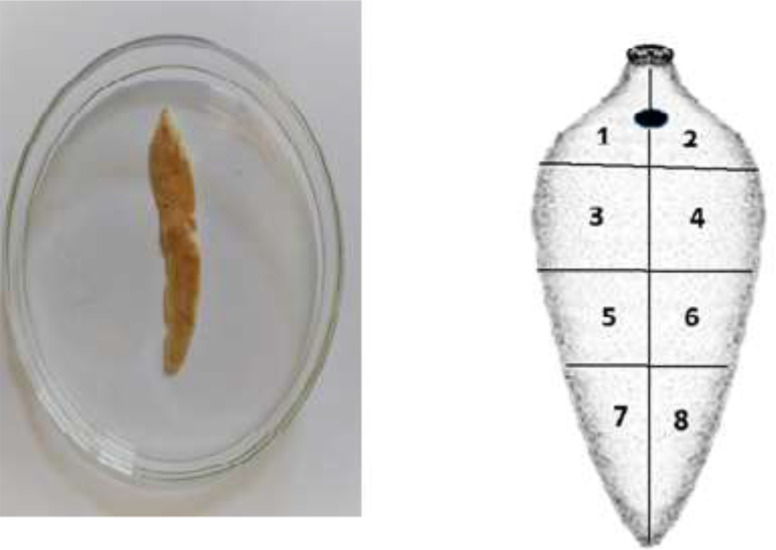
Eight parts of tegumental surface of the worms

With regard to the measurements of spines in the both types (Table 1) the length size, was concluded as the only different parameter with regards to the larger ones in *F. hepatica*. To attain the most definite conclusion of fasciolid species differentiation using the present findings, more increasing in sample size of the adult and immature worms will however elevate the confidence value. Practically, the spines in tissue sections taken from a certain part of the worm are morphologically similar in shape with those in sections from the counterpart region of the same species topographically (Fig. 4). The present paper reveals the capability of a non-molecular tool utilized in species identification between *F. hepatica* and *F. gigantica.*

## Conclusion

Recent advancement in molecular techniques is significantly revolutionized taxonomical studies regarding species identification. Trying to introduce a practical means of species identification, preferably a non-molecular tool seems remarkably innovative meanwhile. In present study, following the morphological studies proved by RFLP-PCR technique, tissue sections were performed to find a reliable non-molecular means for species identification. Morphological characteristics of tegumental spines of *Fasciola* species in tissue sections considered as the main attained result in studied liver flukes herein. To enrich our findings, Scanning Electron Micrographs has been also implemented for the samples. In this study, two types of tegumental spines, pointed and molar shapes have been identified addressing to distinguish *F. hepatica* and *F. gigantica* species respectively. The current study has illustrated a hidden reliable morphological character of *Fasciola* liver fluke in tissue sections which has not been enough concentrated through helminthological publications so far.

## Ethical considerations

Ethical issues (Including plagiarism, informed consent, misconduct, data fabrication and/or falsification, double publication and/or submission, redundancy, etc.) have been completely observed by the authors.
